# Differences in stewardship strategies between hospitals performing well and poorly on a risk-adjusted metric for post-discharge antibiotic use

**DOI:** 10.1017/ice.2024.237

**Published:** 2025-04

**Authors:** Daniel J. Livorsi, James A. Merchant, Hyunkeun Cho, Matthew Bidwell Goetz, Bruce Alexander, Brice Beck, Michihiko Goto

**Affiliations:** 1 Center for Access and Delivery Research and Evaluation, Iowa City Veterans Affairs Health Care System, Iowa City, IA, USA; 2 Division of Infectious Diseases, University of Iowa Carver College of Medicine, Iowa City, IA, USA; 3 Department of Biostatistics, University of Iowa, Iowa City, IA, USA; 4 VA Greater Los Angeles Healthcare System, Los Angeles, CA, USA; 5 David Geffen School of Medicine, University of California, Los Angeles, CA, USA

## Abstract

Stewardship processes were compared across 123 hospitals that differed on a risk-adjusted post-discharge antibiotic use metric. Low-performing hospitals were less likely than high-performing hospitals to report routine interactions between their stewardship physician and pharmacist(s) (OR 0.12, 95% CI 0.03–0.55) and to have local antibiotic-prescribing guidelines (OR 0.21, 95% CI 0.05–0.93)

## Introduction

Antibiotic overuse at hospital discharge is common and increases the risk of antibiotic-related adverse events.^1–3^ It is unclear which antibiotic stewardship strategies are effective at improving antibiotic prescribing at this transition of care. In this mixed-methods study, we compared how hospital characteristics differed based on hospital performance on a risk-adjusted metric for post-discharge antibiotic use.

## Methods

### Data sources

We used the Veterans Health Administration (VHA) Corporate Data Warehouse to collect data on covariates for our risk-adjusted metric, including patient demographics, comorbidities, discharge diagnoses of infection, and inpatient and outpatient antibacterial (hereafter “antibiotic”) use.^4^ Post-discharge antibiotics were oral antibiotics dispensed from the outpatient pharmacy during the discharge period.^5^

We also used data from a mandatory antibiotic stewardship survey of all VHA hospitals. The survey was administered by the VHA’s Healthcare Analysis and Information Group (HAIG). Hospitals were required to respond to the survey between October 20, 2020 and November 10, 2020.

### Patients and hospitals

We included all discharges from VHA acute care that occurred 6 months before/after the deadline for completing the HAIG survey (May 10, 2020 to May 10, 2021). We excluded patients who died while hospitalized, were transferred to another hospital, or were transferred to a post-acute-care facility, as any further antibiotic therapy would be dispensed by that facility and not the VHA outpatient pharmacy (Supplemental Table 1). Patients who received ≥ 30 days of post-discharge oral antibiotics were also excluded because these longer courses likely reflected either (a) chronic antibiotic suppression, (b) antibiotic prophylaxis, or (c) treatment for deep-seated infections. Because some facilities may prefer to treat deep-seated infections with outpatient intravenous antibiotics, which we cannot capture, we did not want to unfairly penalize facilities that more commonly use oral therapy in these cases.

### Statistical analysis

Based on our previously described methods, we built a zero-inflated negative binomial mixed model with two random intercepts for each hospital, one for the count component of the model, the other for the zero-inflated component of the model, to predict post-discharge oral antibiotic exposure and duration.^4^ Using the predicted random intercepts, hospitals were categorized into four groups. High-performing hospitals had negative random intercepts for both components; after adjusting for fixed effects, these hospitals prescribed antibiotics less frequently at discharge and used shorter antibiotic courses. Low-performing hospitals had positive random intercepts for both components; these hospitals prescribed antibiotics more frequently at discharge and used longer antibiotic courses. Intermediate-performing hospitals had a positive random intercept for one component but a negative random intercept for the other.

Once hospitals were categorized into these groups, we compared how their responses differed on certain questions of the HAIG survey. Specific survey questions (Table 1) were chosen to align with strategies of the ROAD Home Framework.^6^ Depending on variables being compared, collinearity between responses was assessed by Pearson’s correlation (all pairwise comparisons <.4), Cohen’s kappa (all comparisons <.3), and the correlation ratio (all comparisons <.3); all collinearity was deemed weak. Next, differences in survey responses across the four groups were assessed using a one-way analysis of variance test in the case of continuous variables and a chi-square goodness of fit test in the case of categorical variables. We then used a multinomial logistic regression model with the four-level post-discharge antibiotic grouping variable as the outcome and the associated processes as predictor variables. All tests were performed at the alpha = .05 level; all data processing and modeling were conducted with R version 4.4.0 (R Core Team, 2024).

**Table 1.** Questions from the VHA mandatory antibiotic stewardship survey that were used for our analysis and how they map to the Reducing Overuse of Antibiotics at Discharge Framework^a^

ROAD Home Tier	HAIG survey question	How responses were operationalized for the analysis
Tier 1	Within the last year, has the facility/HCS’s AS team provided education regarding stewardship principles to inpatient providers?	Yes vs. no
Tier 1	Does the facility/HCS utilize clinical pathways (i.e., antibiotic therapy guidelines, order entry templates) for specific inpatient conditions?	Yes for community-acquired pneumonia, skin and soft tissue infections, and urinary tract infections vs. not answering yes for all 3 conditions
Tier 1	How often do your stewardship Pharmacy and Provider Champions interact regarding stewardship activities?	Daily or several times a week vs. weekly, monthly or less frequently than monthly
Tier 2	Number of inpatient antibiotics managed by prospective audit for continued use at day 3	1–10 (see footnote a)
Tier 2	Number of inpatient antibiotics that require prior approval or must meet local criteria for use	1–10 (see footnote a)
Tier 2	Does the facility/HCS perform a time-out (around day 2–4) for one or more antibiotics and/or classes of antibiotics?	Yes vs. no
Tier 2	Are there automatic stop orders in place for antibiotic duration?	Yes vs. no
Tier 3	How often, at the day of discharge, does your inpatient AS team systematically review patient-level use of one or more antibiotics and/or classes of antibiotic (i.e., perform prospective audit) to determine total duration of therapy?	Always (5+ times/week) or usually (3–4 times/week) vs. sometimes (1–2×/week), seldom (less than once per week) or never
Tier 3	Does the facility/HCS AS team regularly review average length of therapy of persons receiving antibiotics as a metric?	Yes vs. no

Abbreviations: AS, antibiotic stewardship; HCS, healthcare system; ROAD, Reducing Overuse of Antibiotics at Discharge; VHA, Veterans Health Administration.

a This variable ranges from 1 to 10 and captures whether certain antibiotics were managed by restrictions or prospective audit-and-feedback (PAF). Each additional antibiotic managed with one of these strategies increased the score by 1, except as noted: vancomycin (intravenous), daptomycin, oral/IV linezolid (each formulation = 0.5 points), piperacillin-tazobactam, cefepime, ceftazidime, anti-pseudomonal carbapenems, ertapenem, ciprofloxacin + levofloxacin oral/IV (each 0.5), and moxifloxacin oral/IV (each 0.5).

### Ethics

The institutional Review Board of the University of Iowa and Iowa City Veterans Health Care System approved this study.

## Results

There were 396,909 patient admissions across the 123 hospitals. At the hospital level, the median number of qualifying discharges per month was 233.8 (IQR 98.3–364.4); 17.2% received post-discharge antibiotics with a median duration of 6 days (IQR 4–10).

Based on the risk-adjusted metric for post-discharge antibiotic use, 37 (30.1%) hospitals were high-performing, 22 (17.9%) were low-performing, and the other 64 (52.0%) were intermediate-performing (Supplemental Figure 1).

All hospitals responded to the HAIG survey. Compared to low-performing sites, high-performing sites were significantly more likely to be high complexity (62.2% vs. 18.2%; *p* = .003), have more beds (median 89 vs. 35, *p* = .011), and have an ID consultation service (91.9% vs. 54.5%; *p* < .001) [Table 2]. The two stewardship processes associated with metric performance were (1) more frequent interactions between stewardship champions (70.3% vs. 22.7% at high and low-performing sites, respectively; *p* = .001) and (2) education of inpatient providers on stewardship principles within the past year (97.3% vs. 77.3% at high and low-performing sites, respectively; *p* = .038).

**Table 2.** Characteristics of 123 VHA medical centers, stratified by their performance on the risk-adjusted metric for post-discharge antibiotic use

Hospital characteristic	Total	High-performing (n = 37)	Intermediate, group 1 (n = 36)^a^	Intermediate, group 2 (n = 28)^a^	Low-performing (n = 22)	*p*-value
High complexity facility, n (%)^b^	60 (48.8%)	23 (62.2%)	22 (61.1%)	11 (39.3%)	4 (18.2%)	0.003
Acute-care beds, median (IQR)	74 (35–109)	89 (43–113)	93 (64–118)	47 (26–85)	35 (24–78)	0.011
FTEs per 100 beds, median (IQR)^c^	1.3 (0.9–2.2)	1.2 (0.9–2.3)	1.2 (0.8–1.7)	1.7 (0.8–2.5)	1.7 (1.2–2.4)	0.355
ID consultation service, n (%)	101 (82.1%)	34 (91.9%)	33 (91.7%)	22 (78.6%)	12 (54.5%)	<.001

Antibiotic stewardship process	Total	High-performing (n = 37)	Intermediate, group 1 (n = 36)^a^	Intermediate, group 2 (n = 28)^a^	Low-performing (n = 22)	*p*-value
AS team interacts several times per week, n (%)^d^	72 (58.5%)	26 (70.3%)	21 (58.3%)	20 (71.4%)	5 (22.7%)	0.001
Automatic antibiotic stop orders, n (%)	99 (80.5%)	34 (91.9%)	26 (72.2%)	21 (75.0%)	18 (81.8%)	0.158
Local guidelines, n (%)^e^	49 (39.8%)	19 (51.4%)	14 (38.9%)	12 (42.9%)	4 (18.2%)	0.091
Discharge audits ≥ 3x/week, n (%)^f^	67 (54.5%)	24 (64.9%)	16 (44.4%)	17 (60.7%)	10 (45.5%)	0.237
Education of providers, n (%)	112 (91.1%)	36 (97.3%)	32 (88.9%)	27 (96.4%)	17 (77.3%)	0.038
AS team tracks average LOT n (%)	35 (28.5%)	13 (35.1%)	7 (19.4%)	7 (25.0%)	8 (36.4%)	0.378
# inpt antibiotics managed by PAF, mean (SD)^g^	1.6 (2.5)	1.9 (2.7)	1.6 (2.0)	1.5 (2.7)	1.5 (2.8)	0.948
# inpt antibiotics that are restricted, mean (SD)^g^	4.5 (2.6)	4.7 (2.4)	5.0 (2.2)	4.5 (3.0)	3.5 (2.7)	0.160
Inpt antibiotic timeout, n (%)	57 (46.3%)	17 (45.9%)	18 (50.0%)	13 (46.4%)	9 (40.9%)	0.928

Abbreviations: IQR, interquartile range; FTE, full-time equivalent; ID, Infectious Disease; inpt, inpatient; SD, standard deviation; VHA, Veterans Health Administration.

a Intermediate-performing hospitals had a negative random intercept for one component of the model and a positive random intercept for the other component.

b High-complexity facilities were categorized as the first (1a) or second (1b) highest tier of the VHA’s five-level complexity ranking system. Hospitals are scored according to their patient population, clinical services (e.g. intensive care unit [ICU] and surgery services), and education and research. A score of 1a is the most complex while a score of 3 is the least complex.

c Stewardship FTEs could be assigned to a physician, pharmacist, and/or advanced practice provider.

d This survey question was dichotomized to compare stewardship physicians and stewardship pharmacists who interact daily or several times a week versus teams that interact weekly, monthly or less frequently than monthly.

e A positive response indicates that the hospital had local treatment guidelines for three common conditions: community-acquired pneumonia, skin and soft tissue infections, and urinary tract infections.

f A positive response indicates that the hospital adheres to the following process at least 3 times per week: the inpatient antibiotic stewardship team systematically reviews patient-level use of one or more antibiotics and/or classes of antibiotics (i.e., performs prospective audit-and-feedback) to determine total duration of therapy at the day of discharge.

g This variable ranges from 1 to 10 and captures whether certain antibiotics were managed by restrictions or prospective audit-and-feedback (PAF). Each additional antibiotic managed with one of these strategies increased the score by 1, except as noted: vancomycin (intravenous), daptomycin, oral/IV linezolid (each formulation = 0.5 points), piperacillin-tazobactam, cefepime, ceftazidime, anti-pseudomonal carbapenems, ertapenem, ciprofloxacin + levofloxacin oral/IV (each 0.5), and moxifloxacin oral/IV (each 0.5).

In the multinomial logistic regression model of stewardship processes, low-performing hospitals were less likely than high-performing hospitals to (a) report interactions between their stewardship physician and stewardship pharmacist(s) every day or several times per week (OR 0.12, 95% CI 0.03–0.55), and (b) use clinical pathways for common infections (OR 0.21, 95% CI 0.05, 0.93). (Supplemental Table 2).

## Discussion

Our findings suggest that routine stewardship physician-pharmacist collaboration and local antibiotic-prescribing guidelines may be important to reducing antibiotic overuse at discharge. Physician engagement likely provides credibility to initiatives to reduce antibiotic overuse at discharge while local guidelines provide an easy reference for determining duration of therapy. Both components are recommended in the CDC’s Core Elements for hospital-based stewardship programs.^7^

Despite this recommendation, national survey data shows there are gaps in using these strategies. Only 59% of VHA hospitals reported that physician and pharmacist champions met several times a week, and 63% of US acute-care facilities had programs co-led by both a pharmacist and physician.^8^ Local guidelines for pneumonia, urinary tract infections and skin and soft tissue infections were only present at 40% of VHA hospitals. In a survey of all US hospitals, 80% reported having local treatment guidelines, but the question did not specify which infections the guidelines cover.

Our analysis did not identify additional strategies that were associated with better performance on the risk-adjusted metric for post-discharge antibiotic use. Though surprising, this finding is in line with prior work. A mixed-methods study across 39 Michigan hospitals demonstrated that there are multiple pathways to decreasing antibiotic overuse at discharge.^9^ In the Michigan study, the only strategy consistently associated with less post-discharge antibiotic use was reviewing antibiotic prescriptions prior to discharge, but many hospitals were successful at discharge stewardship without using this specific approach.

While the HAIG survey had a 100% response rate, its use for our analysis was a potential weakness. Survey responses may not have been accurate or may have only reflected processes in place at the time of the survey. Furthermore, the survey did not assess how well a stewardship process was implemented and sometimes only asked if a process was used at its most basic level. These limitations could have restricted our ability to identify whether additional strategies were associated with less post-discharge antibiotic use. Future studies should more precisely measure processes to better understand how these are associated with differences in hospital performance at this transition of care.

In conclusion, our mixed-methods study showed that frequent collaboration between the stewardship physician and stewardship pharmacist and local antibiotic-prescribing guidelines were associated with less antibiotic use at discharge. While there may be many ways to reduce antibiotic overuse at this transition of care, these two features seem to be important strategies for success.

## Supporting information

Livorsi et al. supplementary material Livorsi et al. supplementary material

## Supplementary material

The supplementary material for this article can be found at https://doi.org/10.1017/ice.2024.237
