# Supplementary material for: Differences in stewardship strategies between hospitals performing well and poorly on a risk-adjusted metric for post-discharge antibiotic use
Source: Infect Control Hosp Epidemiol. 2025 Feb 12;46(4):427–30. doi: 10.1017/ice.2024.237 (PMC12015618; doi:10.1017/ice.2024.237)
Supplement: Livorsi et al. supplementary material [file S0899823X2400237Xsup001.docx]

**Supplemental Table 1. Study Flowchart**

| **Number of patient-admissions during the study dates (May 10, 2020 – May 10, 2021)** | 465,364 |
| --- | --- |
|  |  |
| **Exclusions** |  |
| Key data is missing | 21 |
| Died while hospitalized | 9,482 |
| Left against medical advice | 10,573 |
| Transferred to another hospital or to a post-acute care facility | 40,609 |
| Missing discharge location | 1,258 |
| Prescribed ≥ 30 days of oral antibiotics at discharge | 6,512 |
|  |  |
| **Number of patient-admissions included in final cohort** | 396,909 |
|  |  |

**Supplemental Figure 1. Risk-adjusted comparison of post-discharge antibiotic prescribing frequency and duration across 123 Veterans Health Administration hospitals, May 2010-May 2021**


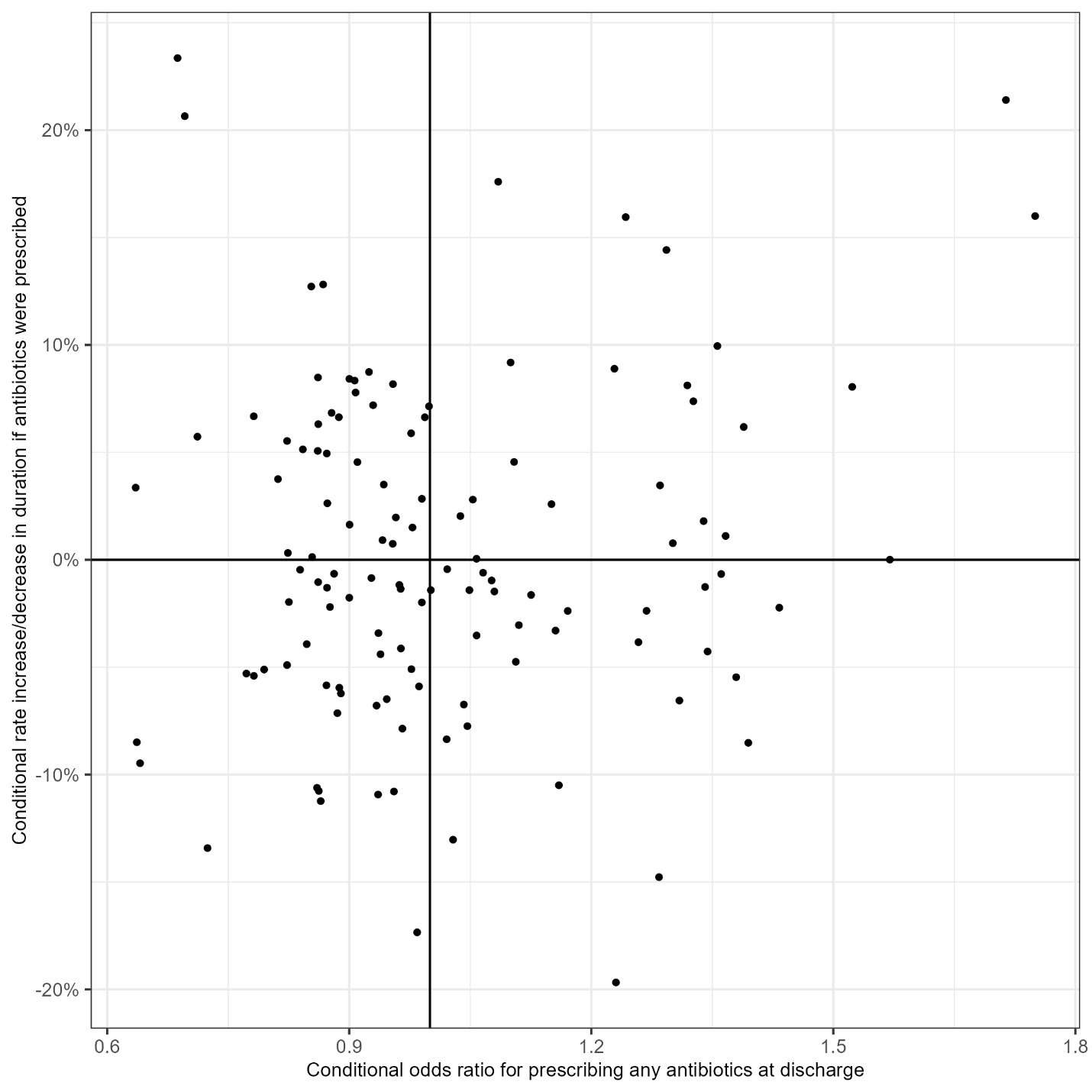


**Figure legend:**

1. Lower left quadrant: High-performing hospitals had less frequent post-discharge antibiotic-prescribing and used shorter post-discharge antibiotic durations.
2. Lower right quadrant: Intermediate-performing hospitals (group 2) had more frequent post-discharge antibiotic-prescribing but used shorter post-discharge antibiotic durations.
3. Upper left quadrant: Intermediate-performing hospitals (group 1) had less frequent post-discharge antibiotic-prescribing but used longer post-discharge antibiotic durations.
4. Upper right quadrant: Low-performing hospitals had more frequent post-discharge antibiotic-prescribing and used longer post-discharge antibiotic durations.

**Supplemental Table 2. Findings from a multinomial logistic regression that compared antibiotic stewardship processes between hospitals performing well on a risk-adjusted metric for post-discharge antibiotic use to all other hospitals**

| **Stewardship process** | **ORs with 95% CI relative to high-performing hospitals** | | |
| --- | --- | --- | --- |
|  | **Intermediate, group 1 (n=36)**^1^ | **Intermediate, group 2 (n=28)**^1^ | **Low-performing (n=22)**^1^ |
| AS team members interact several times per week^2^ | 0.66 (0.21, 2.03) | 1.19 (0.35, 4.05) | 0.12 (0.03, 0.55) |
| Automatic antibiotic stop orders | 0.25 (0.06, 1.08) | 0.27 (0.06, 1.2) | 0.53 (0.09, 3.28) |
| Local antibiotic-prescribing guidelines for common infections | 0.98 (0.34, 2.82) | 0.89 (0.3, 2.63) | 0.21 (0.05, 0.93) |
| At least 1 antibiotic-type audited at discharge at least 3 times per week | 0.56 (0.2, 1.54) | 0.99 (0.34, 2.92) | 0.54 (0.16, 1.83) |
| Education of inpatient providers within the prior year | 0.37 (0.03, 4.27) | 0.85 (0.04, 17.36) | 0.37 (0.03, 4.32) |
| AS team tracks average LOT as a metric | 0.51 (0.16, 1.62) | 0.64 (0.2, 2.01) | 1.8 (0.47, 6.92) |
| Number of inpatient antibiotics managed by PAF^3^ | 0.96 (0.77, 1.19) | 0.91 (0.73, 1.14) | 1.13 (0.87, 1.46) |
| Number of inpatient antibiotics that are restricted^3^ | 1.05 (0.85, 1.29) | 0.94 (0.75, 1.17) | 0.88 (0.69, 1.13) |
| Antibiotic timeout for ≥ 1 inpatient antibiotic-type | 1.12 (0.41, 3.03) | 1.14 (0.4, 3.26) | 0.78 (0.22, 2.71) |

Abbreviation: AS antibiotic stewardship; CI = confidence interval; LOT = length of therapy; OR = odds ratio; PAF = prospective audit-and-feedback.

1. High-performing hospitals (n=40) are the reference group.
2. This survey question was dichotomized to compare stewardship physicians and stewardship pharmacists who interact daily or several times a week versus teams that interact weekly, monthly or less frequently than monthly.
3. The odds ratio reflects the effect on the odds of increasing the number of antibiotics managed with this strategy by a single unit (i.e., managing 10 antibiotics by PAF versus managing only 9 by PAF).
